# A cross-cultural study of autistic traits across India, Japan and the UK

**DOI:** 10.1186/s13229-018-0235-3

**Published:** 2018-11-05

**Authors:** Sophie Carruthers, Emma Kinnaird, Alokananda Rudra, Paula Smith, Carrie Allison, Bonnie Auyeung, Bhismadev Chakrabarti, Akio Wakabayashi, Simon Baron-Cohen, Ioannis Bakolis, Rosa A Hoekstra

**Affiliations:** 1 Institute of Psychiatry, Psychology and Neuroscience, King’s College London, London, UK; 2 Psychology, Ben-Gurion University of the Negev, Beer Sheva, Israel; 3 Autism Research Centre, Department of Psychiatry, University of Cambridge, Cambridge, UK; 4 Department of Psychology, School of Philosophy, Psychology and Language Sciences, University of Edinburgh, Edinburgh, UK; 5 Centre for Autism, School of Psychology and Clinical Language Sciences, University of Reading, Reading, UK; 6 Chiba University, Chiba, Japan

**Keywords:** Autism, Culture, Cross-cultural comparison, Positive predictive values

## Abstract

**Background:**

There is a global need for brief screening instruments that can identify key indicators for autism to support frontline professionals in their referral decision-making. Although a universal set of conditions, there may be subtle differences in expression, identification and reporting of autistic traits across cultures. In order to assess the potential for any measure for cross-cultural screening use, it is important to understand the relative performance of such measures in different cultures. Our study aimed to identify the items on the Autism Spectrum Quotient (AQ)-Child that are most predictive of an autism diagnosis among children aged 4–9 years across samples from India, Japan and the UK.

**Methods:**

We analysed parent-reported AQ-Child data from India (73 children with an autism diagnosis and 81 neurotypical children), Japan (116 children with autism and 190 neurotypical children) and the UK (488 children with autism and 532 neurotypical children). None of the children had a reported existing diagnosis of intellectual disability. Discrimination indices (DI) and positive predictive values (PPV) were used to identify the most predictive items in each country.

**Results:**

Sixteen items in the Indian sample, 15 items in the Japanese sample and 28 items in the UK sample demonstrated excellent discriminatory power (DI ≥ 0.5 and PPV ≥ 0.7), suggesting these items represent the strongest indicators for predicting an autism diagnosis within these countries. Across cultures, good performing items were largely overlapping, with five key indicator items appearing across all three countries (can easily keep track of several different people’s conversations, enjoys social chit-chat, knows how to tell if someone listening to him/her is getting bored, good at social chit-chat, finds it difficult to work out people’s intentions). Four items indicated potential cultural differences. One item was highly discriminative in Japan but poorly discriminative (DI < 0.3) in the UK and India, and a further item had excellent discrimination properties in the UK but poorly discriminated in the Indian and Japanese samples. Two additional items were highly discriminative in two cultures but poor in the third.

**Conclusions:**

Cross-cultural overlap in the items most predictive of an autism diagnosis supports the general notion of universality in autistic traits whilst also highlighting that there can be cultural differences associated with certain autistic traits. These findings have the potential to inform the development of a brief global screening tool for autism. Further development and evaluation work is needed.

**Electronic supplementary material:**

The online version of this article (10.1186/s13229-018-0235-3) contains supplementary material, which is available to authorized users.

## Background

Autism spectrum disorders (ASD), henceforth ‘autism’, are neurodevelopmental conditions, characterised by difficulties with social interaction and communication, unusually repetitive and restricted behaviours and interests and sensory hyper-sensitivity [1]. Despite a considerable amount of research into autism [2], the majority of studies have been conducted in Western, higher income countries [3–6]. Consequently, assumptions surrounding the epidemiology, diagnosis and treatment of autism have not been adequately tested across different cultures and socioeconomic settings.

A diagnosis of autism is based on the behavioural characteristics of an individual. Though core autism characteristics are believed to be universal, there is preliminary evidence to suggest that cultural differences may exert subtle influence over the expression, identification and/or reporting of symptomatology [5, 7–9]. Culturally specific stigmas, norms and priorities may mask or emphasise relative distinctions between autistic traits and typically developing behaviours [3, 7, 9]. For example, previous work validating screening measures in Japan reported that parent judgements of whether their child is interested in their peers do not correlate with autism in Japanese communities as it does in the US [10]. If this example reflects a true cultural difference, such disparities could reflect a relative higher peer interest for Japanese children with autism, a relative lower peer interest for the Japanese typically developing children or that the salience of these symptoms is weaker for Japanese parents. Consequently, the profile of autism symptoms as measured by parent report may not be globally consistent [5, 7].

Tools developed for screening autism are increasingly being used outside their original cultural context [11, 12], with the majority developed in Europe and North America [13, 14]. Due to the emphasis on behavioural symptoms of such tools, if the presentation, salience and reporting of autistic characteristics are not globally consistent, this could impact the ability to use tools developed in one culture (typically the West) in other countries [15]. Developing new screening tools requires extensive resources and effort that may not be feasible for lower income countries. Existing Western screening tools have been translated into other languages [10, 16–20] but not always without difficulties [6, 21, 22], and validation studies of these screening tools in other cultures have typically examined overall mean group differences, rather than item-level analyses [16, 18–20, 23]. Moreover, the previous literature has often focused on toddlers [21, 24, 25]. However, children with autism without intellectual disability are less likely to exhibit salient symptoms at the preschool age and often only receive a diagnosis in mid-late childhood [26–28]. This is particularly problematic since behavioural expectations of children in different countries may differ according to age [29], suggesting that findings on toddler screening measures may neither be translatable to school-aged children, nor necessarily be equivalent across cultures.

There is thus a need for cross-cultural research exploring screening tools for this age group. An important consideration, particularly when aiming to develop a short screening tool, is whether the autistic traits that best predict an autism diagnosis are similar across different cultures. Initial research exploring such ‘key indicators’ has been conducted using the Autism Spectrum Quotient (AQ), a 50-item open-access and free to use questionnaire developed in the UK, adapted for different ages and validated in several languages [19, 24, 30–37]. Researchers developed shorter versions for different age groups (AQ-10) by examining which items best discriminate between cases and controls in UK samples, identifying ten highly predictive items [38]. The AQ-10 exhibited high test accuracy properties and internal consistency and is as effective as the original questionnaire in identifying high-risk autism cases across a range of different ages [38]. However, this analysis has so far only been conducted within a UK sample.

This study aimed to contribute towards a greater understanding of expression and recognition of childhood autism symptoms across cultures by identifying key indicator items across three distinct cultural settings: the UK, Japan and India.

## Methods

### Study sample

The sample from India has been described previously [23]. In brief, participants were recruited from Delhi and Kolkata, using Hindi and Bengali translations of the AQ-Child respectively. Children with a formal autism diagnosis were recruited from not-for-profit organisations in both cities that provide support for people with autism and their families. Typically developing children were recruited from mainstream schools and the general population through word of mouth. Overall, 75 children with autism and 81 typically developing children between the ages of four and eight were recruited from both locations. No children had a reported existing diagnosis of comorbid intellectual disability. Information on the AQ-Child was provided by either parent.

The sample of Japanese participants has not previously been reported; the data collection was coordinated through Chiba University in Japan. Children with a formal autism diagnosis were recruited through special education schools for children with developmental disorders in Tokyo and the surrounding area, typically developing children via mainstream schools. Overall, 116 children with autism and 190 typically developing children between the ages of four and nine were recruited. No children had a comorbid diagnosis other than autism, including no diagnoses of intellectual disability. The AQ-Child was completed by the child’s mother in all instances.

The UK sample was collected by the Autism Research Centre (ARC) at the University of Cambridge. Children with autism were recruited from the ARC’s volunteer database and typically developing children through an epidemiological study of social and communication skills recruited via mainstream primary schools. Overall, the sample consisted of 488 children with autism and 532 typically developing children. The participants included in the current study partly overlaps with the sample reported in previous studies [32, 38]. In contrast to these previous studies, the current project only used data from children aged 4–9 years who resided in the UK. Since the publication of the previous studies, additional data from UK children with ASD has been collected through the volunteer database; these new data are also included in the current study. Further details on the methodology employed in the data collection in the three countries are presented in Table 1.

**Table 1:** Inclusion criteria, recruitment and collection methods of the samples from UK, Japan and India

	UK	Japan	India
Inclusion criteria	*All* Aged 4–9 years Lives in UK No diagnosed ID No siblings in the study	*All* Aged 4–9 years Lives in Tokyo No diagnosed ID No siblings in the study	*All* Aged 4–9 years Lives in Kolkata or Delhi No diagnosed ID Primary language Hindi (if in Delhi) or Bengali (if in Kolkata) No visual, hearing, motor, neurological or mental health disorder No siblings in the study
	*Cases* Diagnosed by recognised clinical service, according to DSM-IV^a^ or DSM-5^b^ criteria.	*Cases* Diagnosis confirmed by school and/or clinic Diagnosis by DSM-IV^a^/ICD-10^c^ No additional diagnosis other than ASD	*Cases* Diagnosis by DSM-IV^a^/ICD-10^c^
	*Controls* No neurodevelopmental disorder	*Controls* No diagnosable condition	*Controls* No formal diagnosis of any mental health condition
Autism recruitment	Via ARC’s volunteer database	Special education schools for children with developmental disorders	Not-for-profit organisations providing support for people with ASD
Control recruitment	Mainstream schools in Cambridgeshire, UK	Mainstream schools in Tokyo	Mainstream schools in Kolkata and Delhi, general population
AQ-Child method of completion	Cases online; controls pen and paper	Pen and paper	Pen and paper
Informant	Either parent	Mothers	Either parent

*ARC* Autism Research Centre, University of Cambridge, *DSM* Diagnostic and Statistical Manual of Mental Disorders, *ICD* International Statistical Classification of Diseases and Related Health Problems, *UK* United Kingdom

^a^DSM-IV [48]

^b^DSM-5 [1]

^c^ICD-10 [49]

### Autism Spectrum Quotient (AQ-Child)

The Autism Spectrum Quotient (AQ-Child) [32] consists of 50 statements relating to autistic traits, where parents indicate on a 4-point Likert scale whether they definitely disagree, slightly disagree, slightly agree or definitely agree with each statement. The AQ includes items assessing a range of autism-characteristic domains, including attention switching, attention to detail, communication, social skills and imagination. The AQ-Child has previously been translated into Japanese [19], Hindi and Bengali [23], with all three versions exhibiting similar psychometric properties to the original [32]. Translation involved blind back translation and multiple cycles of translations until all parties reached consensus. Further details can be found in the respective validation papers [19, 23].

### Statistical analyses

Statistical analyses were conducted with the use of Stata 14.2. AQ item scores were converted from the Likert format into binary scoring for the purpose of these analyses in line with previous work [38]. Relevant items were inverse scored so that a score of 1 indicated the presence of an autistic trait and a score of 0 a negative response.

We randomly split the samples from each country into a derivation and validation sample (Table 2; [38]). Discrimination indices (DI) for each item were calculated using the derivation samples by subtracting the percentage of controls who scored 1 (false positives) from the percentage of cases who scored 1 (true positives). Positive predictive values (PPV) were calculated for each item using the validation samples by dividing the number of true positives by the total number of positives (cases and controls scoring 1).

**Table 2:** Descriptive statistics of the study sample for each country

	Control derivation sample	Autism derivation sample	Control validation sample	Autism validation sample	Total
Japan					
*n*	88	65	102	51	306
Sex					
Female	37	8	60	11	116
Male	51	57	42	40	190
Mean age in years (SD)	7.74 (0.10) (*n* = 88)	7.55 (0.16) (*n* = 65)	7.88 (0.09) (*n* = 102)	7.82 (0.19) (*n* = 51)	
India					
*n*	36	42	45	33	156
Sex					
Female	9	3	12	0	24
Male	9	19	11	16	55
Missing	18	20	22	17	77
Mean age in years (SD)	6.24 (0.87) (*n* = 34)	5.11 (1.09) (*n* = 40)	6.14 (0.24) (*n* = 45)	6.69 (0.27) (*n* = 33)	
UK					
*n*	269	241	263	247	1020
Sex					
Female	152	44	143	42	381
Male	117	197	120	205	639
Mean age in years (SD)	8.84 (0.81)	6.26 (1.65)	8.76 (0.88)	6.49 (1.66)	

*n* Number of participants, *SD* standard deviation

In order to identify a list of key indicator items most predictive of an autism diagnosis within each country, all items per country with a DI ≥ 0.5 (in line with Allison et al.’s previous paper with a UK-based sample [38]) and PPV ≥ 0.7 were selected. Receiver Operating Characteristic (ROC) curves were calculated and compared for these key indicator items and the original 50 items for each country. Optimal cut-offs were determined using the highest percentage correctly classified as guidelines. The area under the curve (AUC) indicates overall predictive validity, with AUC > 0.90 indicating excellent validity. Recommended sensitivity and specificity for developmental screening measures is 70–80% [39]. Cronbach’s Alpha was calculated for each measure with a value of > 0.80 indicating excellent internal consistency. Independent *t* tests were used to assess whether the key indicator items exhibited the expected difference between cases and controls, and Pearson correlations were calculated between key indicator items and AQ-50 total scores for each country.

The relative discrimination properties of all AQ-50 items were compared cross-culturally using the following criteria: DI ≥ 0.5 and PPV ≥ 0.7 = ‘excellent’ discrimination, DI ≥ 0.3 = ‘acceptable’ discrimination and DI < 0.3 = ‘poor’ discrimination [38, 40]. Any item that had ‘excellent’ discrimination in at least one country but ‘poor’ in the other(s) was considered to represent a potential cultural difference. In the UK dataset, there was a significant age difference between controls and cases (see Table 2 and the ‘Results’ section). Therefore, an additional sensitivity analysis was run on the UK dataset to examine whether this age difference could account for the findings.

## Results

Children’s demographic characteristics are summarised in Table 2. There were no age differences between cases and controls in the Japanese and Indian samples; in the UK, the autism group was younger than the control group (*p* < .001) (Table 2).

DI and PPV analyses for each item are summarised in Table 3, with a summary of case/control responses per country for each item included in Additional file 1. Inspection of the DI and PPV values revealed 16 items for the Indian sample with DI ≥ 0.5 and PPV ≥ 0.7 (cells labelled with ‘a’ in Table 3), indicating that these items provided excellent differentiation between autism cases and controls. Similarly, 15 AQ-Child items for the Japanese sample and 28 items for the UK sample surpassed the excellent item performance thresholds (in the middle and right-hand columns of Table 3).

**Table 3:** Item discrimination indices and PPV for each of the 50 items in the AQ across India, Japan and UK

	India		Japan		UK	
AQ item summary	DI	PPV	DI	PPV	DI	PPV
1. Prefers to do things with others rather than alone	.06^c^	.66^c^	.38^b^	.56^b^	.43^b^	.75^b^
2. Prefers to do things the same way over and over again	.52^b^	.60^b^	.54^b^	.59^b^	.62^a^	.70^a^
3. Finds it very easy to create a picture in her/his mind	.67^a^	.94^a^	.45^b^	.89^b^	.55^a^	.81^a^
4. Gets absorbed in one thing and loses sight of other things	.29^c^	.59^c^	.40^b^	.49^b^	.32^b^	.60^b^
5. Notices small sounds when others do not	.20^c^	.46^c^	.35^b^	.61^b^	.52^b^	.68^b^
6. Notices house numbers or similar strings of information	-.25^c^	.33^c^	.37^b^	.80^b^	.30^b^	.61^b^
7. Has difficulty understanding rules for polite behaviour	.58^a^	.78^a^	.44^b^	.96^b^	.80^a^	.89^a^
8. Can easily imagine what characters in a story look like	.86^a^	1^a^	.44^b^	.64^b^	.67^a^	.93^a^
9. Fascinated by dates	-.22^c^	.22^c^	.19^c^	.66^c^	.16^c^	.62^c^
**10. Can easily keep track of different conversations**	**.57** ^**a**^	**.89** ^**a**^	**.51** ^**a**^	**.76** ^**a**^	**.69** ^**a**^	**.79** ^**a**^
11. Finds social situations easy	.68^a^	.90^a^	.60^b^	.66^b^	.75^a^	.86^a^
12. Tends to notice details that others do not	.08^c^	.36^c^	.32^b^	.49^b^	.24^c^	.56^c^
13. Would rather go to a library than a birthday party	.17^c^	.50^c^	.26^c^	.60^c^	.40^b^	.91^b^
14. Finds making up stories easy	.87^a^	.81^a^	.38^b^	.45^b^	.59^a^	.79^a^
15. Drawn more strongly to people than to things	.39^b^	.50^b^	.36^b^	.49^b^	.55^a^	.74^a^
16. Has strong interests, gets upset if cannot pursue	.30^b^	.56^b^	.53^a^	.81^a^	.36^b^	.63^b^
**17. Enjoys social chit-chat**	**.75** ^**a**^	**.75** ^**a**^	**.52** ^**a**^	**.97** ^**a**^	**.71** ^**a**^	**.90** ^**a**^
*18. When talking, it is not easy to get a word in edgeways*	*.02* ^*c*^	*.31* ^*c*^	*.60* ^*a*^	*.83* ^*a*^	*.17* ^*c*^	*.57* ^*c*^
19. Fascinated	-.03^c^	.44^c^	.39^b^	.81^b^	.20^c^	.66^c^
20. Finds it difficult to work out characters’ feelings in a story	.39^b^	.58^b^	.37^b^	.68^b^	.72^a^	.88^a^
21. Does not particularly enjoy fictional stories	.42^b^	.83^b^	.31^b^	.63^b^	.34^b^	.80^b^
22. Finds it hard to make new friends	.64^a^	.74^a^	.39^b^	.67^b^	.67^a^	.85^a^
23. Notices patterns in things all the time	.10^c^	.57^c^	.24^c^	.63^c^	.37^b^	.66^b^
24. Would rather go to the cinema than a museum	-.24^c^	.36^c^	.44^b^	.63^b^	.28^c^	.68^c^
25. Is not upset if daily routine is disturbed	.13^c^	.45^c^	.34^b^	.67^b^	.63^a^	.78^a^
26. Does not know how to keep a conversation going	.64^b^	.68^b^	.78^a^	1^a^	.86^a^	.92^a^
27. Finds it easy to “read between the lines” in conversation	.47^b^	.81^b^	.85^a^	.84^a^	.61^a^	.76^a^
28. Concentrates more on a whole picture, rather than details	.23^c^	.86^c^	.58^b^	.59^b^	.49^b^	.69^b^
29. Not very good at remembering phone numbers	.03^c^	.32^c^	-.08^c^	.26^c^	-.17^c^	.45^c^
30. Does not usually notice small changes	-.12^c^	.36^c^	-.13^c^	.35^c^	-.09^c^	.42^c^
**31. Knows if someone listening is getting bored**	**.65** ^**a**^	**.72** ^**a**^	**.80** ^**a**^	**.87** ^**a**^	**.66** ^**a**^	**.74** ^**a**^
32. Finds it easy to alternate between different activities	.58^a^	.92^a^	.52^b^	.54^b^	.72^a^	.81^a^
33. Not sure when it is her/his turn to speak on the phone	.48^b^	.62^b^	.52^a^	.93^a^	.69^a^	.84^a^
*34. Enjoys doing things spontaneously*	*.23* ^*c*^	*.50* ^*c*^	*.26* ^*c*^	*.82* ^*c*^	*.57* ^*a*^	*.89* ^*a*^
*35. Often the last to understand the point of a joke*	*.14* ^*c*^	*.54* ^*c*^	*.71* ^*a*^	*1* ^*a*^	*.62* ^*a*^	*.81* ^*a*^
36. Finds it easy to tell how someone feels from their face	.68^a^	.80^a^	.59^b^	.60^b^	.69^a^	.87^a^
37. Can switch back to what they were doing if interrupted	.30^b^	.80^b^	.51^a^	.87^a^	.63^a^	.84^a^
**38. Good at social chit-chat**	**.75** ^**a**^	**.86** ^**a**^	**.73** ^**a**^	**.98** ^**a**^	**.80** ^**a**^	**.90** ^**a**^
39. People say they go on and on about the same thing	.44^b^	.68^b^	.59^a^	.94^a^	.41^b^	.70^b^
40. Enjoyed playing pretend games with others in preschool	.78^a^	.87^a^	.38^b^	.69^b^	.71^a^	.86^a^
41. Likes to collect information about categories of things	-.40^c^	.34^c^	.26^c^	.52^c^	.22^c^	.61^c^
42. Finds it difficult to imagine being someone else	.38^b^	.55^b^	.79^a^	.85^a^	.62^a^	.79^a^
43. Likes to plan any activities s/he participates in carefully	-.51^c^	.25^c^	.08^c^	.30^c^	.18^c^	.56^c^
*44. Enjoys social occasions*	*.23* ^*c*^	*.66* ^*c*^	*.51* ^*a*^	*.87* ^*a*^	*.56* ^*a*^	*.92* ^*a*^
**45. Finds it difficult to work out people’s intentions**	**.50** ^**a**^	**.72** ^**a**^	**.80** ^**a**^	**.83** ^**a**^	**.63** ^**a**^	**.76** ^**a**^
46. New situations make him/her anxious	.61^b^	.59^b^	.50^b^	.59^b^	.45^b^	.65^b^
47. Enjoys meeting new people	.40^b^	.82^b^	.25^c^	.51^c^	.49^b^	.84^b^
48. Is good at taking care not to hurt other people’s feelings	.60^a^	.79^a^	.41^b^	.61^b^	.73^a^	.88^a^
49. Not very good at remembering people’s date of birth	-.26^c^	.27^c^	.19^c^	.42^c^	-.18^c^	.46^c^
50. Finds it easy to play pretend games with children	.73^a^	.93^a^	.36^b^	.63^b^	.69^a^	.89^a^

^a^Key indicator item: excellent item performance (DI ≥ 0.5 and PPV ≥ 0.7); ^b^item performed acceptably (DI ≥ 0.3); ^c^item performed poorly (DI < 0.3) Bold text: ‘Universal’ key indicator item with excellent performance across all three countries. Italics: ‘Cultural Difference’ item with variable item performance across countries

### Psychometric properties

Internal consistency was very high for both the India AQ-16 (α = 0.94) and AQ-50 (α = 0.92). The AUC for both versions indicated excellent validity (AUC > 0.90). The AQ-16 and AQ-50 correlated strongly (*r* = 0.89, *p* < .001). At a cut-off point of 5 on the AQ-16, sensitivity was 0.96, specificity was 0.97 and the proportion of correctly classified cases was 0.97. Internal consistency was very high for both the Japanese AQ-15 (α = 0.95) and AQ-50 (α = 0.95). The AUC for both versions indicated excellent validity (AUC > 0.90), and both versions correlated strongly with each other(*r* = 0.95, *p* < .001). At a cut-off point of 12 on the AQ-15, sensitivity was 0.96, specificity was 0.96 and proportion correctly classified was 0.92. Internal consistency was very high for both the UK AQ-28 (α = 0.97) and UK AQ-50 (α = 0.96). The AUC for both versions indicated excellent validity (AUC > 0.90). There was a significant correlation between the AQ-28 and AQ-50 (*r* = 0.97, *p* < .001). At a cut-off point of 14 on the AQ-28, sensitivity was 0.98, specificity was 0.97 and proportion correctly classified was 0.97.

### Cross-cultural comparisons

Five items were identified to be universal key indicators, as they were consistently excellent at discriminating between children with autism and controls in all three countries (see bold items in Table 3). In a social group, s/he can easily keep track of several different people’s conversations; s/he enjoys social chit-chat; s/he knows how to tell if someone listening to him/her is getting bored; s/he is good at social chit-chat and s/he finds it difficult to work out people’s intentions. There were an additional 23 items that performed excellently or acceptably across all three countries.

Four items were identified as indicating potential cultural differences (see items in italics in Table 3). Item 34 (‘S/he enjoys doing things spontaneously’) had excellent discrimination properties in the UK, but discriminated poorly in the Indian and Japanese samples. In contrast, item 18 (‘When s/he talks, it isn’t always easy for others to get a word in edgeways’) performed well in Japan, but poorly in the UK and India. A further two items (35, ‘S/he is often the last to understand the point of a joke’, and 44, ‘S/he enjoys social occasions’) were found to perform poorly in India whilst exhibiting excellent predictive value in the UK and Japan. Further information on how cases and controls in each country responded to the AQ items is available in Additional file 1: Tables S1–S3.

A subgroup analysis restricting the age group to 7–9 years for cases and controls in the UK sample, indicated that age differences between cases and controls in the full UK sample did not explain the pattern of results (Additional file 1: Table S4).

## Discussion

This study aimed to identify which items on the AQ-Child were most predictive of an autism diagnosis among children from India, Japan and the UK. Sixteen items in the Indian sample, 15 in the Japanese sample and 28 items in the UK sample demonstrated high discriminant and predictive ability of ASD cases, excellent psychometric properties and similar sensitivity and specificity values to the original AQ-50. This suggests that at least within cultures, it is possible to adapt existing measures into psychometrically sound brief tools that successfully differentiate children with and without autism.

When comparing the ‘key indicator’ items across cultures, our findings suggest that there is substantial overlap in the items most predictive of an autism diagnosis cross-culturally. Overall, 28 items were found to have acceptable or excellent discrimination properties in all three countries. This suggests that a number of autistic traits are consistently expressed, salient for parents and thus reliably identified and reported across different countries. This provides support for the position that screening measures developed in one country can indeed be used in different cultures. Five items were identified to be consistently excellent at discriminating between children with autism and controls in all three countries and identified as universal key indicators. However, it should be noted that two of these universal items (item 17; s/he enjoys social chit-chat and item 38; s/he is good at social chit-chat) are similarly worded and therefore may be overlapping measurements of the same aspect of behaviour.

The present study also identified four autistic traits that may represent cultural differences. Item 34 (s/he enjoys doing things spontaneously) was a highly predictive item in the UK sample, but not in Japan or India. In the UK, two-thirds of the autism children in the derivation sample were reported to not enjoy spontaneity (in line with autism symptomatology). This ratio was much reduced in the Indian and Japanese samples, where only around 30% of the children with autism were reported to not enjoy spontaneity. By contrast, control children across all three countries were reported to enjoy spontaneity at similar levels (91–97%), suggesting that this difference is specific to the autism group. Cross-cultural studies show that societies differ in their tolerance for uncertainty. For instance, Japan is characterised as a highly uncertainty avoidant society, whereas India and the UK score much lower on uncertainty avoidance [41]. It is possible that as a result of Japanese society’s tendency towards reducing uncertainty, any spontaneous activity is more structured in Japan than in the other cultures, resulting in relatively few children objecting to spontaneous activities. Indian children with autism also appear more accepting of spontaneity that could reflect the prevalence of an authoritarian parenting style in India, resulting in a general reduction in spontaneity across diagnostic groups and so accounting for the reduced predictive power of this item [42]. Alternatively, these differences may be due to linguistic variation rather than a cultural difference: in the Japanese translation of the AQ-Child, the meaning of item 34 was perceived ambiguously by parents and so had to be clarified with a supplemental explanation in addition to the original question [19]. In the supplemental explanation, more emphasis was placed on the meaning of spontaneous as ‘doing something on your own initiative, without suggestions from others’, rather than on ‘doing something without much prior planning’. Similarly, the terms used in the Bengali and Hindi translations of the AQ-Child for ‘spontaneous’ are more common in written than in spoken language. Therefore, these differences in response patterns may reflect a lack of familiarity or ambiguity for parents interpreting the question.

Item 18 (when s/he talks, it is not always easy for others to get a word in edgeways) has strong predictive properties in the Japan sample but not in India or the UK. As expected from a highly predictive item, this item is endorsed (suggesting the presence of the autistic trait) in a larger proportion of the cases (64%) and very few controls (3%) in Japan. In contrast, although endorsed for a large proportion of UK cases (70%), it is also reported in a large proportion of UK controls (53%). For India, the proportion of children for whom it is reported are very similar for both cases (61%) and controls (63%). While lack of qualitative research or cognitive interviewing data prevents us from drawing strong inferences on the causes of these differences, we speculate that parents in the UK and India may have interpreted the item to mean their child was very chatty. While excessive chatting by children is culturally acceptable in the UK and India, the stronger emphasis in Japanese society on social conformity [9, 43–45], politeness and respect for elders may make this characteristic much less acceptable and/or more salient to the reporting parents in Japan.

Items 35 (s/he is often the last to understand the point of a joke) and 44 (s/he enjoys social occasions) were both highly discriminative in the UK and Japan samples but not in India. Although these may be indicative of cultural differences, the smaller size of the Indian sample leads us to interpret these with caution. Moreover, these questions may represent a translation issue: in the versions for India, both ‘joke’ and ‘social occasion’ were translated using more formal language.

### Strengths and limitations

The comparatively smaller number of key indicator items in the India and Japan samples (*n* = 15 and *n* = 16, respectively) in comparison to that of the UK sample (*n* = 28) may reflect the smaller size of the samples for Japan and India compared to the UK. Alternatively, it may indicate that cross-cultural differences generally limit the discriminating power of certain items when the instrument is used outside of the UK culture in which it was originally developed. Moreover, our three samples have come from different research studies, and therefore, subtle differences exist in their sampling characteristics and recruitment procedures. While in all three countries ASD diagnoses were made by a qualified professional using DSM-IV criteria, the exact diagnostic procedures may have varied both within and across country. No data were available on ethnicity, specific IQ information and socio-economic status; all of which may have influenced the results. Additionally, given the vast regional and cultural differences that exist in India, our findings based on relatively small urban population samples may not generalise across all Indian cultures and contexts, particularly rural areas which were not sampled in this study [46]. In all three countries, the autism samples were purposely selected, rather than derived from a population based survey and may therefore not be fully representative of the population of children with autism in each country. In India and Japan, children with autism were recruited from special schools; this sample may represent a subset of autistic trait profiles within the countries and the most predictive items reported in this study may not be as sensitive to more subtle presentations in the community [47]. This highlights the importance of future studies using population-based samples; although this is challenging in low resource contexts.

Across all three countries, data in clinical samples were collected in children in whom autism had previously been diagnosed. This may have resulted in enhanced awareness of parents of their child’s autistic traits and thus increased likelihood of endorsement on corresponding autistic traits. It will be imperative for the development of effective screening tools that future studies explore cross-cultural differences in parent-reports prior to clinical autism diagnoses. It will also be important for comparisons to be conducted in the discrimination of children with ASD and other neurodevelopmental disorders, as this is the more informative contrast for clinicians.

A strength of this study is the exclusion of children with reported diagnoses of intellectual disability, resulting in a more homogenous group of children who are more likely to be left undiagnosed until this primary school age. However, it would also be important to confirm that any measure was equally effective across autism severity, intelligence level and age in each cultural setting. Any global screening initiative would also need to explore any cultural differences in the expression or latent structure of autistic symptomatology in this age group.

Evaluating the utility of the five universal items as a brief screener was beyond the scope of this paper as this would require a different type of psychometric evaluation on a multi-country population-based sample of participants, and we do not recommend use of these items in the place of current screening tools on the basis of these results. However, our findings are informative for the future development of a global screening tool for autism for early-mid childhood, the age when children with autism without intellectual disability are likely to still remain undetected and without formal diagnosis. We identified five items that show consistently excellent performance across all three cultures, suggesting these items hold promise as universal key indicators of autism. This study also identified four items suggesting subtle cultural differences, indicating that researchers should not assume that all autistic traits are equally salient across all cultures. An alternative explanation for the subtle cultural differences identified in this study is the semantic differences in the items concerned. In addition, some of the differences may be of socio-economic rather than cultural origin. To further explore whether the semantics or interpretation of items may be constraining their discriminating abilities and to identify any unique socio-economic or cultural nuances not currently captured by the AQ items, qualitative research (e.g. using cognitive interviews and focus groups) is needed.

## Conclusions

Our analyses have demonstrated that taking the most discriminating items from the AQ-Child from three countries results in psychometrically sound brief measures that correctly classify children with autism and typically developing controls. Items with good discriminating power were, to a large extent, universal across the UK, Japan and India samples, but there were also some potential cultural differences. These findings suggest that five items included in the AQ-50 have consistent excellent power to discriminate autism from control children across three distinct cultures and thus hold promise as cross-cultural key indicators for autism. Additional research is needed to further advance our understanding of the cross-cultural nature of autism symptomatology before a ‘universal’ screening instrument for autism can be derived.

## Additional file

Additional file 1: Supplementary results including breakdown of case/control response proportions by country (**Tables S1-S3**) and sensitivity analysis exploring influence of age in the UK sample (**Table S4**). (DOCX 51 kb)

## Abbreviations

AQ-Child: Autism Spectrum Quotient-Child Version
ARC: Autism Research Centre, University of Cambridge
ASD: Autism spectrum disorder
AUC: Area under the curve
DI: Discrimination indices
DSM-5: Diagnostic and Statistical Manual of Mental Disorders—5th edition
DSM-IV: Diagnostic and Statistical Manual of Mental Disorders—4th edition
ICD-10: International Statistical Classification of Diseases and Related Health Problems, tenth edition
ID: Intellectual disability
N: Number of participants
PPV: Positive predictive value
ROC: Receiver Operating Characteristic curve
SD: Standard deviation
UK: United Kingdom
US: United States
