# Supplementary material for: A cross-cultural study of autistic traits across India, Japan and the UK
Source: Mol Autism. 2018 Nov 5;9:52. doi: 10.1186/s13229-018-0235-3 (PMC6217788; doi:10.1186/s13229-018-0235-3)
Supplement: Supplementary file 1 — Supplementary results including breakdown of case/control response proportions by country (Tables S1-S3) and sensitivity analysis exploring influence of age in the UK sample (Table S4). (DOCX 51 kb) [file 13229_2018_235_MOESM1_ESM.docx]

**Table S1: Case/Control breakdown of proportion of responses and discrimination indices per item for data from India**

|  |  | **Cases** | | **Controls** | |  |
| --- | --- | --- | --- | --- | --- | --- |
|  |  | **0** | **1** | **0** | **1** |  |
| **Item** | **Subscale** | **n (%)** | **n (%)** | **n (%)** | **n (%)** | **DI** |
| **01** | Social | 27 (65.85) | 14 (34.15) | 26 (72.22) | 10 (27.78) | 0.06 |
| **02** | Attention Switching | 6 (14.29) | 36 (85.71) | 24 (66.67) | 12 (33.33) | 0.52 |
| **03** | Imagination | 11 (27.50) | 29 (72.50) | 33 (94.29) | 2 (5.71) | 0.67 |
| **04** | Attention Switching | 9 (21. 43) | 33 (78.57) | 18 (50.00) | 18 (50.00) | 0.29 |
| **05** | Attention to Detail | 17 (40.48) | 25 (59.52) | 21 (60.00) | 14 (40.00) | 0.20 |
| **06** | Attention to Detail | 33 (80.49) | 8 (19.51) | 20 (55.56) | 16 (44.44) | -0.25 |
| **07** | Communication | 12 (28.57) | 30 (71.43) | 31 (86.11) | 5 (13.89) | 0.58 |
| **08** | Imagination | 6 (14.29) | 36 (85.71) | 36 (100.00) | 0 (0.00) | 0.86 |
| **09** | Attention to Detail | 36 (85.71) | 6 (14.29) | 23 (63.89) | 13 (36.11) | -0.22 |
| **10** | Attention Switching | 11 (26.19) | 31 (73.81) | 30 (83.33) | 6 (16.67) | 0.57 |
| **11** | Social | 11 (26.19) | 31 (73.81) | 32 (94.12) | 2 (5.88) | 0.68 |
| **12** | Attention to Detail | 16 (38.10) | 26 (61.90) | 14 (38.89) | 22 (61.11) | 0.08 |
| **13** | Social | 30 (71.43) | 12 (28.57) | 31 (88.57) | 4 (11.43) | 0.17 |
| **14** | Imagination | 3 (7.14) | 39 (92.86) | 34 (94.44) | 2 (5.56) | 0.87 |
| **15** | Social | 15 (35.71) | 27 (64.29) | 27 (75.00) | 9 (25.00) | 0.39 |
| **16** | Attention Switching | 8 (19.05) | 34 (80.95) | 17 (48.57) | 18 (51.43) | 0.30 |
| **17** | Communication | 7 (17.07) | 34 (82.93) | 33 (91.67) | 3 (8.33) | 0.75 |
| **18** | Communication | 23 (60.53) | 15 (39.47) | 22 (62.86) | 13 (37.14) | 0.02 |
| **19** | Attention to Detail | 26 (63.41) | 15 (36.59) | 20 (60.61) | 13 (39.39) | -0.03 |
| **20** | Imagination | 18 (43.90) | 23 (56.10) | 30 (83.33) | 6 (16.67) | 0.39 |
| **21** | Imagination | 17 (41.46) | 24 (58.54) | 30 (83.33) | 6 (16.67) | 0.42 |
| **22** | Social | 8 (19.05) | 34 (80.95) | 30 (83.33) | 6 (16.67) | 0.64 |
| **23** | Attention to Detail | 20 (47.62) | 22 (52.38) | 20 (57.14) | 15 (42.86) | 0.10 |
| **24** | Imagination | 20 (54.05) | 17 (45.95) | 10 (30.30) | 23 (69.70) | -0.24 |
| **25** | Attention Switching | 25 (59.52) | 17 (40.48) | 26 (72.22) | 10 (27.78) | 0.13 |
| **26** | Communication | 7 (16.67) | 35 (83.33) | 29 (80.56) | 7 (19.44) | 0.64 |
| **27** | Communication | 17 (41.46) | 24 (58.54) | 32 (88.89) | 4 (11.11) | 0.47 |
| **28** | Attention to Detail | 26 (63.41) | 15 (36.59) | 31 (86.11) | 5 (13.89) | 0.23 |
| **29** | Attention to Detail | 22 (53.66) | 19 (46.34) | 20 (57.14) | 15 (42.86) | 0.03 |
| **30** | Attention to Detail | 18 (42.86) | 24 (57.14) | 11 (30.56) | 25 (69.44) | -0.12 |
| **31** | Communication | 4 (10.26) | 35 (89.74) | 27 (75.00) | 9 (25.00) | 0.65 |
| **32** | Attention Switching | 15 (36.59) | 26 (63.41) | 33 (94.29) | 2 (5.71) | 0.58 |
| **33** | Communication | 13 (34.21) | 25 (65.79) | 28 (82.35) | 6 (17.65) | 0.48 |
| **34** | Attention Switching | 30 (71.43) | 12 (28.57) | 33 (94.29) | 2 (5.71) | 0.23 |
| **35** | Communication | 21 (52.50) | 19 (47.50) | 24 (66.67) | 12 (33.33) | 0.14 |
| **36** | Social | 10 (23.81) | 32 (76.19) | 33 (91.67) | 3 (8.33) | 0.68 |
| **37** | Attention Switching | 27 (64.29) | 15 (35.71) | 34 (94.44) | 2 (5.56) | 0.30 |
| **38** | Communication | 7 (17.07) | 34 (82.93) | 33 (91.67) | 3 (8.33) | 0.75 |
| **39** | Communication | 15 (39.47) | 23 (60.53) | 30 (83.33) | 6 (16.67) | 0.44 |
| **40** | Imagination | 8 (19.51) | 33 (80.49) | 34 (97.14) | 1 (2.86) | 0.78 |
| **41** | Imagination | 30 (73.17) | 11 (26.83) | 12 (33.33) | 24 (66.67) | -0.40 |
| **42** | Imagination | 13 (30.95) | 29 (69.05) | 25 (69.44) | 11 (30.56) | 0.38 |
| **43** | Attention Switching | 29 (74.36) | 10 (25.64) | 8 (22.86) | 27 (77.14) | -0.51 |
| **44** | Social | 30 (71.43) | 12 (28.57) | 34 (94.44) | 2 (5.56) | 0.23 |
| **45** | Social | 8 (19.05) | 34 (80.95) | 25 (69.44) | 11 (30.56) | 0.50 |
| **46** | Attention Switching | 8 (19.05) | 34 (80.95) | 28 (80.00) | 7 (20.00) | 0.61 |
| **47** | Social | 20 (48.78) | 21 (51.22) | 32 (88.89) | 4 (11.11) | 0.40 |
| **48** | Social | 11 (26.19) | 31 (73.81) | 30 (85.71) | 5 (14.29) | 0.60 |
| **49** | Attention to Detail | 30 (73.17) | 11 (26.83) | 16 (47.06) | 18 (52.94) | -0.26 |
| **50** | Imagination | 9 (21.43) | 33 (78.57) | 33 (94.29) | 2 (5.71) | 0.73 |

*0 indicated the absence of the trait, 1 indicated the presence of the trait, DI = discrimination indices, n = number of responses, % = percentage of responses*

**Table S2: Case/Control breakdown of proportion of responses and discrimination indices per item for data from Japan**

|  |  | **Cases (n=65)** | | **Controls (n=88)** | |  |
| --- | --- | --- | --- | --- | --- | --- |
|  |  | **0** | **1** | **0** | **1** |  |
| **Item** | **Subscale** | **n (%)** | **n (%)** | **n (%)** | **n (%)** | **DI** |
| 01 | Social | 28 (43.08) | 37 (56.92) | 71 (80.68) | 17 (19.32) | 0.38 |
| 02 | Attention Switching | 9 (13.85) | 56 (86.15) | 60 (68.18) | 28 (31.82) | 0.54 |
| 03 | Imagination | 32 (49.23) | 33 (50.77) | 83 (94.32) | 5 (5.68) | 0.45 |
| 04 | Attention Switching | 9 (13.85) | 56 (86.15) | 47 (53.41) | 41 (46.59) | 0.40 |
| 05 | Attention to Detail | 23 (35.38) | 42 (64.62) | 62 (70.45) | 26 (29.55) | 0.35 |
| 06 | Attention to Detail | 36 (55.38) | 29 (44.62) | 81 (92.05) | 7 (7.95) | 0.37 |
| 07 | Communication | 35 (53.85) | 30 (46.15) | 86 (97.73) | 2 (2.27) | 0.44 |
| 08 | Imagination | 23 (35.28) | 42 (64.62) | 78 (88.64) | 10 (11.36) | 0.44 |
| 09 | Attention to Detail | 44 (67.69) | 21 (32.31) | 76 (86.36) | 12 (13.64) | 0.19 |
| 10 | Attention Switching | 25 (38.46) | 40 (61.54) | 79 (89.77) | 9 (10.23) | 0.51 |
| 11 | Social | 13 (20.00) | 52 (80.00) | 70 (79.55) | 18 (20.45) | 0.60 |
| 12 | Attention to Detail | 21 (32.31) | 44 (67.69) | 57 (64.77) | 31 (35.23) | 0.32 |
| 13 | Social | 35 (53.85) | 30 (46.15) | 70 (79.55) | 19 (20.45) | 0.26 |
| 14 | Imagination | 22 (33.85) | 43 (66.15) | 63 (71.59) | 25 (28.41) | 0.38 |
| 15 | Social | 11 (16.92) | 54 (83.08) | 47 (53.41) | 41 (46.59) | 0.36 |
| 16 | Attention Switching | 22 (33.85) | 43 (66.15) | 76 (86.36) | 12 (13.64) | 0.53 |
| 17 | Communication | 30 (46.15) | 35 (53.86) | 86 (97.73) | 2 (2.27) | 0.52 |
| 18 | Communication | 24 (36.92) | 41 (63.08) | 85 (96.59) | 3 (3.41) | 0.60 |
| 19 | Attention to Detail | 34 (52.31) | 31 (47.69) | 80 (90.91) | 8 (9.09) | 0.39 |
| 20 | Imagination | 29 (44.62) | 36 (55.38) | 72 (81.82) | 16 (18.18) | 0.37 |
| 21 | Imagination | 35 (53.85) | 30 (46.15) | 75 (85.23) | 13 (14.77) | 0.31 |
| 22 | Social | 29 (44.62) | 36 (55.38) | 73 (82.95) | 15 (17.05) | 0.39 |
| 23 | Attention to Detail | 36 (55.38) | 29 (44.62) | 70 (79.55) | 19 (20.45) | 0.24 |
| 24 | Imagination | 24 (36.92) | 41 (63.08) | 71 (80.68) | 17 (19.32) | 0.44 |
| 25 | Attention Switching | 28 (43.08) | 37 (56.92) | 68 (77.27) | 20 (22.73) | 0.34 |
| 26 | Communication | 14 (21.54) | 51 (78.46) | 88 (100.00) | 0 (0.00) | 0.78 |
| 27 | Communication | 7 (10.77) | 58 (89.23) | 84 (95.45) | 4 (4.55) | 0.85 |
| 28 | Attention to Detail | 8 (12.31) | 57 (87.69) | 62 (70.45) | 26 (29.55) | 0.58 |
| 29 | Attention to Detail | 26 (40.00) | 39 (60.00) | 28 (31.82) | 60 (68.18) | -0.08 |
| 30 | Attention to Detail | 27 (41.54) | 38 (58.46) | 25 (28.41) | 63 (71.59) | -0.13 |
| 31 | Communication | 5 (7.69) | 60 (92.31) | 77 (87.50) | 11 (12.50) | 0.80 |
| 32 | Attention Switching | 7 (10.77) | 58 (89.23) | 55 (62.50) | 33 (37.50) | 0.52 |
| 33 | Communication | 31 (47.69) | 34 (52.31) | 88 (100.00) | 0 (0.00) | 0.52 |
| 34 | Attention Switching | 46 (70.77) | 19 (29.23) | 85 (96.59) | 3 (3.41) | 0.26 |
| 35 | Communication | 19 (29.23) | 46 (70.77) | 88 (100.00) | 0 (0.00) | 0.71 |
| 36 | Social | 12 (18.46) | 53 (81.54) | 68 (77.27) | 20 (22.73) | 0.59 |
| 37 | Attention Switching | 26 (40.00) | 39 (60.00) | 80 (90.91) | 8 (9.09) | 0.51 |
| 38 | Communication | 17 (26.15) | 48 (73.85) | 87 (98.86) | 1 (1.14) | 0.73 |
| 39 | Communication | 25 (38.46) | 40 (61.54) | 86 (97.73) | 2 (2.27) | 0.59 |
| 40 | Imagination | 28 (43.08) | 37 (56.92) | 71 (80.68) | 17 (19.32) | 0.38 |
| 41 | Imagination | 21 (32.31) | 44 (67.69) | 51 (57.95) | 37 (42.05) | 0.26 |
| 42 | Imagination | 10 (15.38) | 55 (84.62) | 83 (94.32) | 5 (5.68) | 0.79 |
| 43 | Attention Switching | 46 (70.77) | 19 (29.23) | 69 (78.41) | 19 (21.59) | 0.08 |
| 44 | Social | 31 (47.69) | 34 (52.31) | 87 (98.86) | 1 (1.14) | 0.51 |
| 45 | Social | 4 (6.15) | 61 (93.85) | 76 (86.36) | 12 (13.64) | 0.80 |
| 46 | Attention Switching | 15 (23.08) | 50 (76.92) | 64 (72.73) | 24 (27.27) | 0.50 |
| 47 | Social | 25 (38.46) | 40 (61.54) | 56 (63.64) | 32 (36.36) | 0.25 |
| 48 | Social | 28 (43.08) | 37 (56.92) | 74 (84.09) | 14 (15.91) | 0.41 |
| 49 | Attention to Detail | 29 (44.62) | 36 (55.38) | 56 (63.64) | 32 (36.36) | 0.19 |
| 50 | Imagination | 27 (41.54) | 38 (58.46) | 68 (77.27) | 20 (22.73) | 0.36 |

*0 indicated the absence of the trait, 1 indicated the presence of the trait, DI = discrimination indices, n = number of responses, % = percentage of responses*

**Table S3: Case/Control breakdown of proportion of responses and discrimination indices per item for data from the UK**

|  |  | **Cases (n=241)** | | **Controls (n=269)** | |  |
| --- | --- | --- | --- | --- | --- | --- |
|  |  | **0** | **1** | **0** | **1** |  |
| **Item** | **Subscale** | **n (%)** | **n (%)** | **n (%)** | **n (%)** | **DI** |
| 01 | Social | 82 (34.0) | 159 (66.0) | 207 (77.0) | 62 (23.0) | 0.43 |
| 02 | Attention Switching | 18 (7.5) | 223 (92.5) | 187 (69.5) | 82 (30.5) | 0.62 |
| 03 | Imagination | 76 (31.5) | 165 (68.5) | 234 (87.0) | 35 (13.0) | 0.55 |
| 04 | Attention Switching | 14 (5.8) | 227 (94.2) | 103 (38.3) | 166 (61.7) | 0.32 |
| 05 | Attention to Detail | 31 (12.9) | 210 (87.1) | 174 (64.7) | 95 (35.3) | 0.52 |
| 06 | Attention to Detail | 51 (21.2) | 190 (78.8) | 138 (51.3) | 131 (48.7) | 0.30 |
| 07 | Communication | 28 (11.6) | 213 (88.4) | 246 (91.4) | 23 (8.6) | 0.80 |
| 08 | Imagination | 63 (26.1) | 178 (73.9) | 251 (93.3) | 18 (6.7) | 0.67 |
| 09 | Attention to Detail | 139 (57.7) | 102 (42.3) | 198 (73.6) | 71 (26.4) | 0.16 |
| 10 | Attention Switching | 14 (5.8) | 227 (94.2) | 201 (74.7) | 68 (25.3) | 0.69 |
| 11 | Social | 22 (9.1) | 219 (90.9) | 225 (84.0) | 43 (16.0) | 0.75 |
| 12 | Attention to Detail | 23 (9.5) | 218 (90.5) | 91 (33.8) | 178 (66.2) | 0.24 |
| 13 | Social | 127 (52.7) | 114 (47.3) | 249 (92.6) | 20 (7.4) | 0.40 |
| 14 | Imagination | 61 (25.3) | 180 (74.7) | 226 (84.0) | 43 (16.0) | 0.59 |
| 15 | Social | 53 (22.0) | 188 (78.0) | 208 (77.3) | 61 (22.7) | 0.55 |
| 16 | Attention Switching | 16 (6.6) | 225 (93.4) | 115 (42.8) | 154 (57.2) | 0.36 |
| 17 | Communication | 43 (17.8) | 198 (82.2) | 240 (89.2) | 29 (10.8) | 0.71 |
| 18 | Communication | 72 (29.9) | 169 (70.1) | 125 (46.5) | 144 (53.5) | 0.17 |
| 19 | Attention to Detail | 94 (39.0) | 147 (61.0) | 160 (59.5) | 109 (40.5) | 0.20 |
| 20 | Imagination | 40 (16.6) | 201 (83.4) | 238 (88.5) | 31 (11.5) | 0.72 |
| 21 | Imagination | 139 (57.7) | 102 (42.3) | 247 (91.8) | 22 (8.2) | 0.34 |
| 22 | Social | 33 (13.7) | 208 (86.3) | 217 (80.7) | 52 (19.3) | 0.67 |
| 23 | Attention to Detail | 58 (24.1) | 183 (75.9) | 163 (60.6) | 106 (39.4) | 0.37 |
| 24 | Imagination | 111 (46.1) | 130 (53.9) | 199 (74.0) | 70 (26.0) | 0.28 |
| 25 | Attention Switching | 47 (19.5) | 194 (80.5) | 223 (82.9) | 46 (17.1) | 0.63 |
| 26 | Communication | 24 (10.0) | 217 (90.0) | 258 (95.9) | 11 (4.1) | 0.86 |
| 27 | Communication | 25 (10.4) | 216 (89.6) | 192 (71.4) | 77 (28.6) | 0.61 |
| 28 | Attention to Detail | 31 (12.9) | 210 (87.1) | 166 (61.7) | 103 (38.3) | 0.49 |
| 29 | Attention to Detail | 112 (46.5) | 129 (53.5) | 78 (29.0) | 191 (71.0) | -0.17 |
| 30 | Attention to Detail | 80 (33.2) | 161 (66.8) | 65 (24.2) | 204 (75.8) | -0.09 |
| 31 | Communication | 14 (5.8) | 227 (94.2) | 192 (71.4) | 77 (28.6) | 0.66 |
| 32 | Attention Switching | 37 (15.4) | 204 (84.6) | 236 (87.7) | 33 (12.3) | 0.72 |
| 33 | Communication | 39 (16.2) | 202 (83.8) | 228 (84.8) | 41 (15.2) | 0.69 |
| 34 | Attention Switching | 82 (34.0) | 159 (66.0) | 245 (91.1) | 24 (8.9) | 0.57 |
| 35 | Communication | 47 (19.5) | 194 (80.5) | 219 (81.4) | 50 (18.6) | 0.62 |
| 36 | Social | 43 (17.8) | 198 (82.2) | 234 (87.0) | 35 (13.0) | 0.69 |
| 37 | Attention Switching | 59 (24.5) | 182 (75.5) | 235 (87.4) | 34 (12.6) | 0.63 |
| 38 | Communication | 22 (9.1) | 219 (90.9) | 240 (89.2) | 29 (10.8) | 0.80 |
| 39 | Communication | 42 (17.4) | 199 (82.6) | 156 (58.0) | 113 (42.0) | 0.41 |
| 40 | Imagination | 34 (14.1) | 207 (85.9) | 228 (84.8) | 41 (15.2) | 0.71 |
| 41 | Imagination | 86 (35.7) | 155 (64.3) | 155 (57.6) | 114 (42.4) | 0.22 |
| 42 | Imagination | 33 (13.7) | 208 (86.3) | 204 (75.8) | 65 (24.2) | 0.62 |
| 43 | Attention Switching | 90 (37.3) | 151 (62.7) | 150 (55.8) | 119 (44.2) | 0.18 |
| 44 | Social | 96 (39.8) | 145 (60.2) | 257 (95.5) | 12 (4.5) | 0.56 |
| 45 | Social | 18 (7.5) | 223 (92.5) | 190 (70.6) | 79 (29.4) | 0.63 |
| 46 | Attention Switching | 18 (7.5) | 223 (92.5) | 141 (52.4) | 128 (47.6) | 0.45 |
| 47 | Social | 85 (35.3) | 156 (64.7) | 228 (84.8) | 41 (15.2) | 0.49 |
| 48 | Social | 33 (13.7) | 208 (86.3) | 232 (86.2) | 37 (13.8) | 0.73 |
| 49 | Attention to Detail | 132 (54.8) | 109 (45.2) | 99 (36.8) | 170 (63.2) | -0.18 |
| 50 | Imagination | 54 (22.4) | 187 (77.6) | 246 (91.4) | 23 (8.6) | 0.69 |

*0 indicated the absence of the trait, 1 indicated the presence of the trait, DI = discrimination indices, n = number of responses, % = percentage of responses*

**Table S4: Item discrimination indices and PPV for each of the 50 items in the AQ across India, Japan and the UK only including UK control group participants aged 7-9 years**

|  | **India** | | **Japan** | | **UK** | |
| --- | --- | --- | --- | --- | --- | --- |
| **AQ Item Summary** | **DI** | **PPV** | **DI** | **PPV** | **DI** | **PPV** |
| 1. Prefers to do things with others rather than alone | .06^c^ | .66^c^ | .38^b^ | .56^b^ | .41^b^ | .49^b^ |
| 2. Prefers to do things the same way over and over again | .52^b^ | .60^b^ | .54^b^ | .59^b^ | .57^b^ | .50^b^ |
| 3. Finds it very easy to create a picture in her/his mind | .67^a^ | .94^a^ | .45^b^ | .89^b^ | .51^b^ | .64^b^ |
| 4. Gets absorbed in one thing and loses sight of other things | .29^c^ | .59^c^ | .40^b^ | .49^b^ | .32^b^ | .38^b^ |
| 5. Notices small sounds when others do not | .20^c^ | .46^c^ | .35^b^ | .61^b^ | .56^b^ | .46^b^ |
| 6. Notices house numbers or similar strings of information | -.25^c^ | .33^c^ | .37^b^ | .80^b^ | .36^b^ | .38^b^ |
| 7. Has difficulty understanding rules for polite behaviour | .58^a^ | .78^a^ | .44^b^ | .96^b^ | .75^a^ | .80^a^ |
| 8. Can easily imagine what characters in a story look like | .86^a^ | 1^a^ | .44^b^ | .64^b^ | .64^a^ | .79^a^ |
| 9. Fascinated by dates | -.22^c^ | .22^c^ | .19^c^ | .66^c^ | .20^c^ | .40^c^ |
| **10. Can easily keep track of different conversations** | **.57^a^** | **.89^a^** | **.51^a^** | **.76^a^** | **.68^a^** | **.60^a^** |
| 11. Finds social situations easy | .68^a^ | .90^a^ | .60^b^ | .66^b^ | .81^b^ | .66^b^ |
| 12. Tends to notice details that others do not | .08^c^ | .36^c^ | .32^b^ | .49^b^ | .25^c^ | .34^c^ |
| 13. Would rather go to a library than a birthday party | .17^c^ | .50^c^ | .26^c^ | .60^c^ | .41^b^ | .82^b^ |
| 14. Finds making up stories easy | .87^a^ | .81^a^ | .38^b^ | .45^b^ | .53^b^ | .60^b^ |
| 15. Drawn more strongly to people than to things | .39^b^ | .50^b^ | .36^b^ | .49^b^ | .52^b^ | .56^b^ |
| 16. Has strong interests, gets upset if can’t pursue | .30^b^ | .56^b^ | .53^a^ | .81^a^ | .42^b^ | .39^b^ |
| **17. Enjoys social chit-chat** | **.75^a^** | **.75^a^** | **.52^a^** | **.97^a^** | **.73^a^** | **.73^a^** |
| 18. When talking, it isn’t easy to get a word in edgeways | .02^c^ | .31^c^ | .60^a^ | .83^a^ | .28^c^ | .37^c^ |
| 19. Fascinated | -.03^c^ | .44^c^ | .39^b^ | .81^b^ | .13^c^ | .42^c^ |
| 20. Finds it difficult to work out characters’ feelings in a story | .39^b^ | .58^b^ | .37^b^ | .68^b^ | .78^a^ | .70^a^ |
| 21. Doesn’t particularly enjoy fictional stories | .42^b^ | .83^b^ | .31^b^ | .63^b^ | .47^b^ | .64^b^ |
| 22. Finds it hard to make new friends | .64^a^ | .74^a^ | .39^b^ | .67^b^ | .69^b^ | .65^b^ |
| 23. Notices patterns in things all the time | .10^c^ | .57^c^ | .24^c^ | .63^c^ | .39^b^ | .44^b^ |
| 24. Would rather go to the cinema than a museum | -.24^c^ | .36^c^ | .44^b^ | .63^b^ | .28^c^ | .40^c^ |
| 25. Is not upset if daily routine is disturbed | .13^c^ | .45^c^ | .34^b^ | .67^b^ | .64^b^ | .60^b^ |
| 26. Doesn’t know how to keep a conversation going | .64^b^ | .68^b^ | .78^a^ | 1^a^ | .78^a^ | .83^a^ |
| 27. Finds it easy to “read between the lines” in conversation | .47^b^ | .81^b^ | .85^a^ | .84^a^ | .64^b^ | .54^b^ |
| 28. Concentrates more on a whole picture, rather than details | .23^c^ | .86^c^ | .58^b^ | .59^b^ | .50^b^ | .46^b^ |
| 29. Not very good at remembering phone numbers | .03^c^ | .32^c^ | -.08^c^ | .26^c^ | -.13^c^ | .24^c^ |
| 30. Doesn’t usually notice small changes | -.12^c^ | .36^c^ | -.13^c^ | .35^c^ | -.14^c^ | .23^c^ |
| 31. Knows if someone listening is getting bored | .65^a^ | .72^a^ | .80^a^ | .87^a^ | .57^b^ | .56^b^ |
| 32. Finds it easy to alternate between different activities | .58^a^ | .92^a^ | .52^b^ | .54^b^ | .68^b^ | .67^b^ |
| 33. Not sure when it’s her/his turn to speak on the phone | .48^b^ | .62^b^ | .52^a^ | .93^a^ | .61^b^ | .69^b^ |
| 34. Enjoys doing things spontaneously | .23^c^ | .50^c^ | .26^c^ | .82^c^ | .63^a^ | .71^a^ |
| 35. Often the last to understand the point of a joke | .14^c^ | .54^c^ | .71^a^ | 1^a^ | .63^b^ | .61^b^ |
| 36. Finds it easy to tell how someone feels from their face | .68^a^ | .80^a^ | .59^b^ | .60^b^ | .70^a^ | .70^a^ |
| 37. Can switch back to what they were doing if interrupted | .30^b^ | .80^b^ | .51^a^ | .87^a^ | .65^b^ | .69^b^ |
| **38. Good at social chit-chat** | **.75^a^** | **.86^a^** | **.73^a^** | **.98^a^** | **.81^a^** | **.75^a^** |
| 39. People say they go on and on about the same thing | .44^b^ | .68^b^ | .59^a^ | .94^a^ | .45^b^ | .45^b^ |
| 40. Enjoyed playing pretend games with others in preschool | .78^a^ | .87^a^ | .38^b^ | .69^b^ | .78^b^ | .66^b^ |
| 41. Likes to collect information about categories of things | -.40^c^ | .34^c^ | .26^c^ | .52^c^ | .23^c^ | .42^c^ |
| 42. Finds it difficult to imagine being someone else | .38^b^ | .55^b^ | .79^a^ | .85^a^ | .64^b^ | .58^b^ |
| 43. Likes to plan any activities s/he participates in carefully | -.51^c^ | .25^c^ | .08^c^ | .30^c^ | .25^c^ | .35^c^ |
| 44. Enjoys social occasions | .23^c^ | .66^c^ | .51^a^ | .87^a^ | .62^a^ | .81^a^ |
| 45. Finds it difficult to work out people’s intentions | .50^a^ | .72^a^ | .80^a^ | .83^a^ | .66^b^ | .54^b^ |
| 46. New situations make him/her anxious | .61^b^ | .59^b^ | .50^b^ | .59^b^ | .44^b^ | .43^b^ |
| 47. Enjoys meeting new people | .40^b^ | .82^b^ | .25^c^ | .51^c^ | .53^b^ | .61^b^ |
| 48. Is good at taking care not to hurt other people’s feelings | .60^a^ | .79^a^ | .41^b^ | .61^b^ | .73^a^ | .70^a^ |
| 49. Not very good at remembering people’s date of birth | -.26^c^ | .27^c^ | .19^c^ | .42^c^ | -.04^c^ | .23^c^ |
| 50. Finds it easy to play pretend games with children | .73^a^ | .93^a^ | .36^b^ | .63^b^ | .67^a^ | .72^a^ |

^a^Key indicator item: excellent item performance (DI ≥ 0.5 and PPV ≥ 0.7) ^b^Item performed acceptably (DI ≥ 0.3) ^c^Item performed poorly (DI < 0.3) **Bold text: ‘Universal’ key indicator item excellent item performance across all 3 countries.** *Italics: ‘Cultural Difference’ item with variable item performance across countries.*
